# Should Hypervascular Incidentalomas Detected on Per-Interventional Cone Beam Computed Tomography during Intra-Arterial Therapies for Hepatocellular Carcinoma Impact the Treatment Plan in Patients Waiting for Liver Transplantation?

**DOI:** 10.3390/cancers16132333

**Published:** 2024-06-26

**Authors:** Haytham Derbel, Athena Galletto Pregliasco, Sébastien Mulé, Julien Calderaro, Youssef Zaarour, Laetitia Saccenti, Mario Ghosn, Edouard Reizine, Maxime Blain, Alexis Laurent, Raffaele Brustia, Vincent Leroy, Giuliana Amaddeo, Alain Luciani, Vania Tacher, Hicham Kobeiter

**Affiliations:** 1 Medical Imaging Department, Henri Mondor University Hospital, 51 Avenue du Marechal de Lattre de Tassigny, 94010 Creteil, Francehicham.kobeiter@aphp.fr (H.K.); 2 Institut Mondor de Recherche Biomédicale, Inserm U955, Team n° 18, 94010 Creteil, France; 3 Faculty of Medicine, University of Paris Est Creteil, 94010 Creteil, France; 4 Laboratory of Pathology, Henri Mondor University Hospital, 94010 Creteil, France; 5 Department of Visceral Surgery, Henri Mondor University Hospital, 94010 Creteil, France; 6 Department of Hepatology, Henri Mondor University Hospital, 94010 Creteil, France

**Keywords:** hepatocellular carcinoma, liver neoplasms, neoplasm recurrence, magnetic resonance imaging, prognosis

## Abstract

**Simple Summary:**

Discovering hypervascular incidentalomas (HVIs) during intra-arterial therapies (IATs) for hepatocellular carcinoma (HCC) is a common condition, but guidelines lack precise management suggestions. This study examines whether to include HVIs in IAT for HCC patients awaiting liver transplantation. A retrospective study analyzed liver-transplanted HCC patients who received TACE or TARE before LT from 2014 to 2018. The study compared HCC detection rates between pre-interventional imaging and per-interventional CBCT and investigated correlations between HVIs and poor prognosis criteria. Results showed higher nodule detection with CBCT and no significant correlations between HVIs and poor prognosis criteria, tumor recurrence, or mortality. Kaplan–Meier analysis found no significant impact of HVIs on recurrence-free, recurrence-related, or overall survival. These data may indicate that the treatment plan during IAT should not be impacted or modified in response to HVI detection in patients awaiting LT.

**Abstract:**

Background: Current guidelines do not indicate any comprehensive management of hepatic hypervascular incidentalomas (HVIs) discovered in hepatocellular carcinoma (HCC) patients during intra-arterial therapies (IATs). This study aims to evaluate the prognostic value of HVIs detected on per-interventional cone beam computed tomography (CBCT) during IAT for HCC in patients waiting for liver transplantation (LT). Material and methods: In this retrospective single-institutional study, all liver-transplanted HCC patients between January 2014 and December 2018 who received transarterial chemoembolization (TACE) or radioembolization (TARE) before LT were included. The number of ≥10 mm HCCs diagnosed on contrast-enhanced pre-interventional imaging (PII) was compared with that detected on per-interventional CBCT with a nonparametric Wilcoxon test. The correlation between the presence of an HVI and histopathological criteria associated with poor prognosis (HPP) on liver explants was investigated using the chi-square test. Tumor recurrence (TR) and TR-related mortality were investigated using the chi-square test. Recurrence-free survival (RFS), TR-related survival (TRRS), and overall survival (OS) were assessed according to the presence of HVI using Kaplan–Meier analysis. Results: Among 63 included patients (average age: 59 ± 7 years, H/F = 50/13), 36 presented HVIs on per-interventional CBCT. The overall nodule detection rate of per-interventional CBCT was superior to that of PII (median at 3 [Q1:2, Q3:5] vs. 2 [Q1:1, Q3:3], respectively, *p* < 0.001). No significant correlation was shown between the presence of HVI and HPP (*p* = 0.34), TR (*p* = 0.095), and TR-related mortality (0.22). Kaplan–Meier analysis did not show a significant impact of the presence of HVI on RFS (*p* = 0.07), TRRS (0.48), or OS (*p* = 0.14). Conclusions: These results may indicate that the treatment plan during IAT should not be impacted or modified in response to HVI detection.

## 1. Introduction

Liver transplantation (LT) is a valuable therapeutic option in the management of hepatocellular carcinoma (HCC), as proposed by the Barcelona Clinic Liver Cancer (BCLC) recommendations for early- and intermediate-stage patients [1,2]. Indications for this complex therapy are growing worldwide due to the increasing incidence of HCC and its excellent long-term outcome for well-selected patients [3]. LT allows the elimination of both tumors and underlying liver disease, which is the leading risk factor for tumor recurrence (TR) in cases of hepatic resection [4]. Transplanted patient outcomes depend on several factors, including patient selection [1,3,5]; tumor biology (i.e., alpha-fetoprotein level) [3,5]; tumor-related features such as size, presence of liver metastasis or multinodularity, vascular invasion, and poor tumor differentiation [4,5,6,7]; tumor metabolic activity on FDG-PET imaging; and response to locoregional bridging therapy [3,4,5,8,9,10,11]. Previous studies have shown some limitations of pre-interventional imaging (PII) staging in accurately predicting TR after LT, mainly regarding the tumor burden estimation and the exhaustive detectability of HCC nodules [5,6,12]. A discrepancy related to these parameters between PII and explanted liver histopathological findings was reported, suggesting the need to reassess the risk of HCC recurrence post-LT [5,13,14]. Intra-arterial therapies (IATs) provide additional per-interventional imaging, and discordances between PII and IAT imaging have been reported in many previous studies [12,15,16,17,18].

IAT, including transarterial chemoembolization (TACE) and, more recently, transarterial radioembolization (TARE), is usually proposed as a locoregional treatment (LRT) for HCC patients awaiting LT [8,19,20,21]. These therapies aim to prevent dropout from the waiting list and to decrease TR [7,8,21,22,23,24]. A recent study by Young et al. [17] investigated the correlation between novel hypervascular foci detected on digital subtracted angiography (DSA) during TACE for HCC and the outcomes of transplanted patients. According to this study, the discordance between PII and per-interventional DSA findings was associated with a higher risk of dropout from the transplant list and TR after transplantation. On the other hand, the superiority of cone beam computed tomography (CBCT) over DSA and magnetic resonance imaging (MRI) in hypervascular lesion detection has been well demonstrated [15,16,25]. To our knowledge, these novel hepatic hypervascular lesions (HVIs), called incidentalomas [26], have never been investigated on CBCT during IAT. These incidentalomas might be related to the natural evolution of HCC disease or the better sensitivity of CBCT. It was proven that dual-phase (DP) CBCT is at least as sensitive as MRI or contrast-enhanced CT for detecting hypervascular nodules during TACE procedures [12,16,27,28,29]. Current IAT guidelines and experts’ recommendations do not specify any specific management of these incidentalomas [30,31,32]. Currently, including an incidentaloma in the treatment plan depends uniquely on the operator’s experience and does not meet any evidence-based argument. In the context of LT, we can assume that per-operative HVI detection could negatively impact the prognosis of transplanted patients and probably lead to a dropout from the waiting list.

This study aimed to evaluate the prognostic value of liver HVI detected on per-interventional CBCT during IAT for HCC in patients waiting for LT.

## 2. Materials and Methods

### 2.1. Study Design and Patient Population

This monocentric observational retrospective study was approved by a local ethics and institutional review board committee (CERIM—2308-375). Because of the retrospective design and the study’s noninterventional nature, the written informed consent requirement was waived.

From January 2013 to December 2018, all consecutive liver-transplanted patients with HCC for whom IAT (TACE or TARE) was performed as a bridging therapy (BT) or downstaging therapy (DT) under dual-phase open trajectory CBCT guidance were included. Criteria for exclusion were (1) patients without PII or liver explant anatomopathological analysis results available in their medical records, (2) patients for whom IAT was performed without per-interventional DP-CBCT imaging or with images hampered by respiratory or metallic artifacts, and (3) patients lost to follow-up. Patients were included in the LT list according to the combination of Milan criteria and alpha-fetoprotein (AFP) score [5,24,33]. IAT was indicated as BT for patients within the Milan criteria when the estimated waiting time was ≥6 months, while DT was aimed to reduce the tumor load and bring patients outside the Milan criteria into the LT eligibility criteria [9,24]. All medical decisions were discussed in a multidisciplinary liver tumor board meeting, including at least a hepatologist, a hepatobiliary surgeon, a pathologist, a liver-subspecialized radiologist, and an interventional radiologist.

### 2.2. Pre-Interventional Imaging

All patients underwent pre-interventional dynamic contrast-enhanced imaging: contrast-enhanced CT and/or multiphase MRI with a pre-intervention delay of less than two months. MRI exams were performed using a 1.5-T system (Avanto; Siemens Healthcare, Erlangen, Germany) or a 3-T system (Skyra; Siemens Healthcare, Erlangen, Germany) equipped with a phased-array torso coil with an 18-channel system. All MR exams followed a standardized imaging protocol detailed in Table 1. The dynamic contrast-enhanced 3D VIBE T1-weighted sequence included four repeated series (bolus-triggered arterial, portal, venous, and late phases) after injection of 0.2 mL/kg gadoterate meglumine (Dotarem; Guerbet, Aulnay-sous-Bois, France) or 0.1 mL/kg gadobenate dimeglumine (Multihance; Bracco Imaging, Milan, Italy) at a rate of 2 mL/s. If necessary, delayed hepatobiliary phase acquisitions were acquired after a mean delay of 90 min following gadobenate dimeglumine injection T1-weighted sequences [34,35,36].

CT exams were acquired on a 64-detector row CT scanner (Discovery CT750 HD system; General Electric Healthcare, Milwaukee, WI). Patients were examined using the following protocol: unenhanced, late-arterial, and portal phase acquisitions after intravenous injection of 1.5 mL/kg of nonionic contrast agent (Iomeron, 350-Iomeprol; Bracco Imaging, Milan, Italy) at an injection rate of 3–4 mL/s. A bolus-tracking technique was performed with automated scan triggering (SmartPrep; General Electric Healthcare, Milwaukee, WI, USA). An elliptic region of interest (ROI) was positioned in the descending thoracic aorta at the diaphragm level, and the threshold enhancement value was set at 100 HU. The late arterial phase was acquired with a delay of 20 s after aortic enhancement threshold timing. CT acquisition and reconstruction parameters included tube current range (mA): 150–650 (mean: 455); tube voltage (kVp): 120; rotation time: 0.7 s; pitch: 1.375; automatic exposure control: Auto mA-Smart mA; noise index: 25; field of view: large body; reconstruction kernel: standard; section thickness: 0.625 mm. Native raw data of the acquired images were reconstructed using model-based iterative reconstruction, and then the reconstructed images were transferred and archived in the institutional picture archiving and communication system.

### 2.3. Per-Interventional Imaging

All IAT procedures were performed in an angiographic suite equipped with a flat panel detector C-arm angiographic system (Allura Xper FD20 and Allura Clarity; Philips Healthcare, Best, The Netherlands) under CBCT guidance. Patients were placed in a supine position on the angiography table. The celiac trunk and then the hepatic artery were catheterized by a 5 French Cobra catheter, and nonselective hepatic digital subtracted angiography was performed initially to display the global arterial anatomy of the liver. The dual-phase open trajectory protocol was applied for all CBCT scans, including two consecutive 5-s C-arm rotations after a single intra-arterial contrast medium injection [25,37,38]. The early scan (5 s after contrast injection) served to identify tumor-feeding arteries, whereas the delayed scan (17 s after contrast injection) displayed parenchymal and tumoral enhancement [25,37,38]. Open trajectory acquisition was used to ensure the maximum coverage of the liver parenchyma. Each rotation acquired 312 frames (60 frames/s) covering a 240° clockwise arc. The flat panel detector displayed an FOV of 250 × 250 × 193 mm with a matrix size of 384 × 384 × 297 pixels. The acquired 3D volumetric CBCT images had an isotropic resolution of 0.65 mm. Intra-arterial contrast medium (iodixanol 320 mg iodine/mL, Visipaque; GE Healthcare AS, Oslo, Norway) was injected following this protocol: (a) for nonselective injection, 20 mL of contrast medium was injected at a rate of 2 mL/s; (b) for selective (hemi-hepatic) injection, 10 mL of contrast medium was injected at a rate of 1 mL/s. Patients were instructed to hold their breath during acquisition with free breathing between the 2 phases to avoid motion artifacts [37,38]. CBCT scan projections were automatically transferred to a dedicated 3D workstation (Xtravision; Philips Medical Systems, Best, The Netherlands) for analysis of multiplanar and 3D reconstruction of acquired data.

### 2.4. Imaging Analysis

PII data, referred to as the “gold standard”, were reviewed in consensus by two abdominal interventional radiologists with eight years of experience (HD and AG). The two readers were blinded to the clinical data, per-interventional imaging data, and explant anatomopathological analysis. HCC tumors were diagnosed on PII data according to the Liver Imaging Reporting and Data System (LI-RADS) criteria [39]. We considered only LI-RADS 5 tumors planned for IAT according to multidisciplinary board meeting decisions.

Second, per-interventional CBCT images were reviewed for each patient in consensus by both readers to compare the detectability of all ≥1 cm hypervascular nodules included in the field of view on either arterial or late CBCT phases by reference to the PII. For TARE group patients, we evaluated CBCT scans acquired during work-up procedures.

Following this evaluation, patients were categorized into two groups: (1) *incidentaloma+* group, in whom at least one HVI was detected on per-interventional CBCT, and (2) *incidentaloma−* group, with no HVI detected on per-interventional CBCT compared with the PII.

### 2.5. Anatomopathological Analysis

Following each LT, the liver explant was analyzed by a 15-year-experienced liver-subspecialized pathologist (JC). After formalin fixation, explants were macroscopically examined and systematically dissected into 10 mm sections. The number, size, and location of all visible nodules were recorded; then, these nodules were sampled and fixed in paraffin for histological investigation. Active tumoral lesions and focal benign nodules were identified. As part of the study, anatomopathological analysis was performed retrospectively based on detailed pathological reports of each liver explant. Three histopathological criteria associated with poor prognosis (HPP) were recorded for each explant: capsular effraction, macroscopic and/or microscopic vascular invasion, and liver metastasis [5,7,14].

### 2.6. Patient Follow-Up and Prognosis Evaluation

Our institutional post-LT follow-up protocol included immediate postoperative monitoring until patient recovery and regular surveillance every six months. Biological monitoring included liver function tests and serum alpha-fetoprotein (AFP) levels [5,40]. Radiological monitoring consisted of a multiphasic contrast-enhanced CT scan performed every six months during the first two years and then annually afterward. TR was tracked by serum AFP levels and chest and abdominal CT scans. When TR was suspected, liver MRI and ^18^FDG PET-CT were performed. TR was confirmed by combining imaging criteria or pathologic data after percutaneous biopsy.

### 2.7. Statistical Analysis

Data collection and statistical analysis were performed using Microsoft Excel 365 (Microsoft Corporation, Redmond, WA, USA) and SPSS Version 25.0 (SPSS Inc., Chicago, IL, USA). Categoric variables are expressed herein as frequencies. The normal distribution of continuous variables was verified using the Shapiro–Wilk test. Normally distributed continuous variables are expressed as the means ± SDs and ranges. Other continuous variables are expressed as medians, Q1 (25%) and Q3 (75%).

The number of detected nodules on per-interventional CBCT and PII was compared using the nonparametric Wilcoxon test. Similarly, the number of detected nodules on per-interventional CBCT, of all nodules, and of active HCC nodules on liver explants were compared using the Wilcoxon test. Bivariate analysis using the Spearman test assessed the correlation between the number of detected nodules on per-interventional imaging and the number of active HCC nodules.

The correlation between the presence of HVI on per-interventional CBCT and HPP was investigated using Fisher’s exact test.

Finally, recurrence-free survival (RFS), overall survival (OS), and tumor-recurrence-related survival (TRRS) were evaluated using Kaplan–Meier analysis and compared using the log-rank test.

A two-sided *p*-value of 0.05 was considered statistically significant for all statistical tests.

## 3. Results

### 3.1. Patient Characteristics

**Figure 1:**
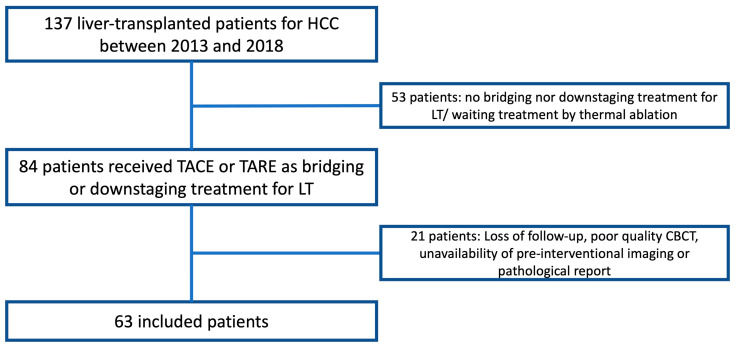
Study flowchart showing patient selection criteria.

During the study period, 137 patients were liver-transplanted for HCC in our institution. Among them, 84 patients (61.3%) received IAT as BT or DT for LT. Twenty-one patients were excluded from the study because of loss of follow-up (*n* = 3), poor quality of CBCT (*n* = 8), and unavailability of preoperative imaging or pathological reports (*n* = 10). Finally, a total of 63 patients (mean age: 59 ± 7 years, sex ratio: 50/13) were included in the study, as detailed in the flow chart (Figure 1). The epidemiologic, clinical, and biological characteristics of the included patients at the time of LT are detailed in Table 2.

### 3.2. Imaging Data Analysis

Contrast-enhanced CT was performed in 24 patients, and MRI was performed in 39 patients as PII, with a mean pre-IAT delay of 35 ± 14 days (4–58 days).

IAT was performed as BT in 49 patients (77.8%) and as DT in 14 patients (22.2%), with a median pre-LT delay of 3 months (1, 6). Conventional TACE was performed in 55 patients (87.3%) and TARE in 8 patients (12.7%).

In all included patients, the overall nodule detection rate of per-interventional CBCT was superior to that of PII (median at 3 [2, 5] vs. 2 [1, 3], respectively, *p* < 0.001).

**Figure 2:**
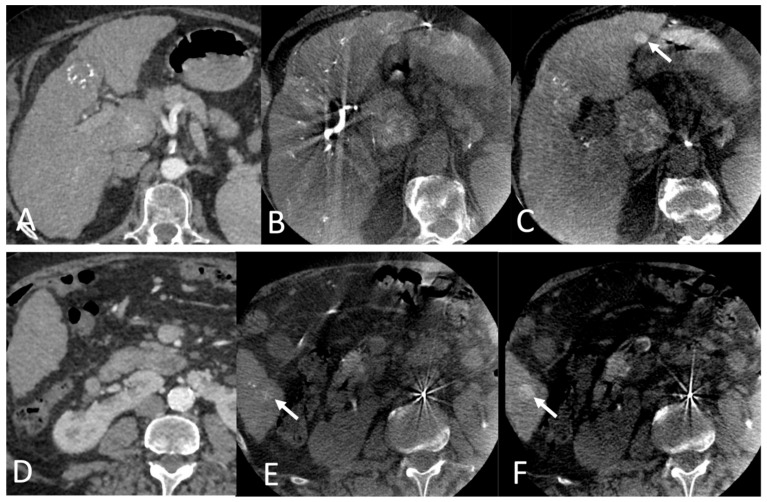
(**A**,**D**): CT axial images at the late arterial phase. (**B**,**C**,**E**,**F**): CBCT axial images at the arterial (**B**,**E**) and parenchymal (**C**,**F**) phases: Hypervascular incidentaloma (white arrows) detected in segments III and VI by per-interventional CBCT during a TACE performed one month after the CT scan. Note the better visibility of hypervascular nodules in the parenchymal phase of dual-phase CBCT.

Thirty-six patients (57,1%) showed HVI on per-interventional CBCT compared with PII (median number of nodules at 4 [3, 8] vs. 2 [1, 5], respectively, *p* < 0.001) (Figure 2). In this group, the median number of HVIs was 2 [1, 3] and the mean size of the largest target was 24.6 ± 13 mm on per-interventional CBCT vs. 23.8 ± 13 mm on PII (*p* = 0.22). HVIs were distributed in the right lobe in 28 patients (44.4%), in the left lobe in 21 patients (33.3%), and in both lobes in the remaining 14 patients (22.2%). Due to retrospective aspect of the study and the absence of comprehensive guidelines, HVIs were subjectively targeted during IAT in only 13 of 34 patients (38.2%), with a mean targeted nodule/all nodule ratio of 0.92 ± 13 (0.5–1). In the *incidentaloma*− group (*n* = 27, 42.9%), both imaging modalities detected no significant difference in the number of nodules (2 [1, 3], *p* = 0.07). In this group, the mean size of the largest target was 30.4 ± 12 mm vs. 29.8 ± 12 mm (*p* = 0.08) (Table 3).

### 3.3. Correlation with Histopathological Features

A median of 3 (1, 5) nodules were detected on histopathological analysis of liver explants with a median of 2 (1, 4) active HCC nodules (*p* < 0.001). There was no significant difference between the median number of detected nodules on per-interventional CBCT and those on liver explants (3 [2, 5] vs. 3 [2, 5], respectively, *p* = 0.97) or active HCC nodules (*p* = 0.1). The number of detected nodules on per-interventional CBCT and that of active HCC nodules on liver explants were significantly correlated (*p* = 0.01) according to the Spearman test. In the “*incidentaloma+*” *group*, no significant difference was noted between the median number of detected nodules on per-interventional CBCT and those on liver explants, whether HVI was targeted (*p* = 0.066) or not (0.84).

**Figure 3:**
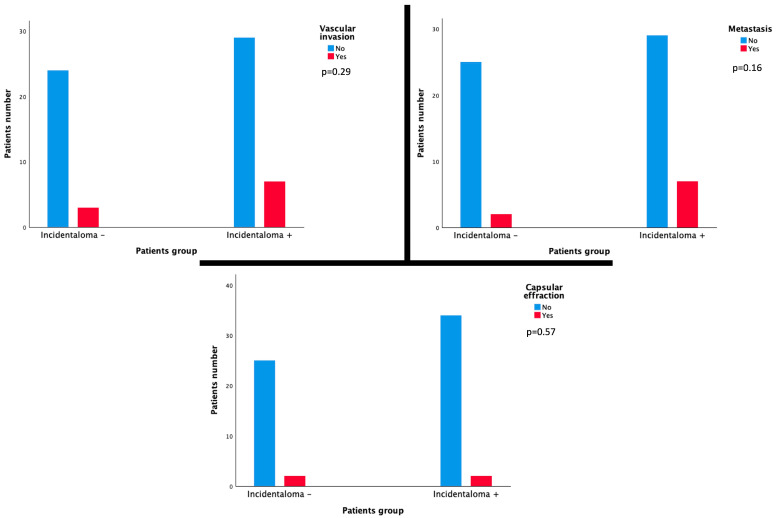
Histograms showing no significant correlation between the presence of HVI on per-interventional CBCT and each HPP: capsular effraction *p*-value = 0.57, vascular invasion *p*-value = 0.29, and presence of liver metastasis *p*-value = 0.16.

HPP was found in 25 patients (39.1%) and was detailed as follows: capsular effraction in 4 patients (6.3%), microvascular invasion in 10 patients (15%), and the presence of liver metastases in 10 patients (15.9%). No HPP was associated with the presence of HVI on per-interventional CBCT: capsular effraction *p* = 0.57; microvascular invasion *p* = 0.29, and presence of liver metastasis *p* = 0.16 (Figure 3). Targeting HVI during IAT did not affect the presence of HPP in liver explants (*p* = 0.17).

In the BT and DT subgroups, HPP was found in 18 patients (36%) and 7 patients (50%), respectively, with no significant difference between the two subgroups (*p* = 0.53).

### 3.4. Correlation with Patient Prognosis

The included patients were followed up for a mean period of 28 ± 15 months (0–49). TR was noted in 12 patients (19%), and the OS rate was 76.2% (48 of 63 patients) at the data collection date. The tumor-recurrence-related mortality was 11.1% (7 of 63 patients) and represented 46.6% of all-cause mortality (7 of 15 patients).

According to the therapeutic intention of the IAT (BT or DT), there was no significant difference between the two subgroups concerning TR (*p* = 0.44) and survival (*p* = 0.29).

**Figure 4:**
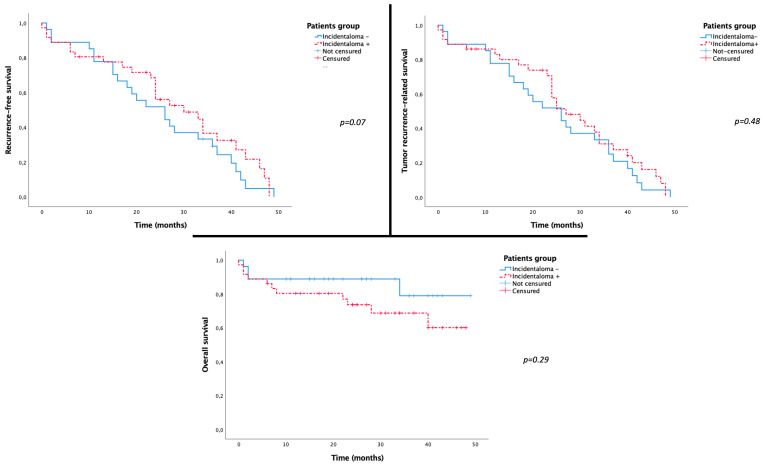
Kaplan–Meier curves showing no significant difference between the two groups *incidentaloma+* and *incidentaloma−* regarding RFS (*p* = 0.07), TRRS (0.48), and OS (*p* = 0.14).

Neither TR nor tumor-recurrence-related mortality was correlated with the presence of HVI on per-interventional CBCT (*p* = 0.1 and *p* = 0.22, respectively). Kaplan–Meier analysis did not show any significant difference between the two groups regarding RFS (*p* = 0.07), TRRS (0.48), or OS (*p* = 0.14) (Figure 4). Targeting HVI during IAT did not affect TR (*p* = 0.21) or OS (*p* = 0.72).

Analysis performed in each subgroup according to the therapeutic intention of IAT (BT or DT) showed no significant difference in terms of TR or OS between patients incidentaloma+ or − (Table 4).

## 4. Discussion

This study confirms the higher sensitivity of CBCT in detecting hepatic hypervascular nodules compared with cross-sectional dynamic enhanced imaging (MRI and CT), as demonstrated by previous studies [12,16,29,41]. This superiority of CBCT can be explained by several factors: mainly, the higher contrast-to-noise ratio and spatial resolution of CBCT [12,42], as well as a better sensitivity for HCC detection of the dual-phase acquisition–single contrast injection protocol [12,29,38,43,44]. Additionally, a mean delay of 35 ± 14 days between PII and IAT can probably explain the higher number of hypervascular nodules on the per-interventional CBCT, considering the natural evolution of the disease. The number of detected nodules on per-interventional CBCT was not significantly different from those found on histopathological analysis (*p* = 0.97) or active HCC nodules (*p* = 0.1). Moreover, the number of detected nodules on per-interventional imaging and that of active HCC nodules on liver explants were significantly correlated (*p* = 0.01). These data suggest that HVIs detected on CBCT are effective tumoral nodules. Nevertheless, this finding should be qualified without a comprehensive radio-pathological correlation regarding the anatomical distribution of nodules.

As histopathological criteria are validated prognostic factors predicting TR after LT [7], we hypothesized that more aggressive HCC with HPP tends to spread rapidly with a higher risk of intrahepatic metastasis and thus of incidentaloma appearance [24,45]. The present study did not confirm this hypothesis because no significant correlation was shown between the presence of HVI on per-interventional CBCT and HPP on liver explant analysis. This finding can be explained partly by the therapeutic effect of IAT on HCC nodules, with modifications of the tumor biology and microenvironment [24]. Post-therapeutic tumor necrosis induces histological modifications, probably leading to an underestimation of HPP (23,24,46). Rodriguez et al. and Zori et al. demonstrated that TARE decreases microvascular invasion by radiation-induced apoptosis of endothelial cells [46,47]. In the present study, HVI was targeted in 38.2% of patients during IAT, with a targeting ratio near 1, partially explaining the absence of a correlation between HVI and HPP.

Interestingly, the present study did not show any significant correlation between the presence of HVI on per-interventional CBCT and poor outcomes after LT. TR and tumor-recurrence-related mortality were not significantly higher in the *incidentaloma+* group than in the other group (*p* = 0.1 and *p* = 0.22, respectively). Similarly, RFS, TRRS, and OS were not correlated with the presence of HVI in the Kaplan–Meier analysis. The absence of a correlation, despite the slight positive trend in the *incidentaloma+* group, could be explained by several factors: first, the therapeutic effect of IAT performed as a bridging/downstaging approach before LT (HVI targeted in 38.2% of *incidentaloma+* patients) may disguise the theoretical impact of HVI. In fact, a prospective study including 200 HCC patients who underwent LT after TACE with drug-eluting beads proved that there was no significant difference in terms of posttransplant OS and TR between patients with tumors initially exceeding the Milan criteria and downstaged after TACE and those within the Milan criteria in patients undergoing DEB-TACE [48]. This fact was confirmed as well in TARE by Salem et al. [49], who showed, through a 1000-patient series, no significant difference in terms of RFS between patients bridged versus those downstaged or within versus beyond the Milan criteria. The present study showed a significant difference between the total number of nodules on histopathological analysis and that of HCC-active nodules, suggesting an effective impact of performing IAT. Meanwhile, the relatively low rate of targeted nodules (38.2% in the *incidentaloma+* group) could explain why the effective prognostic impact of the tumoral load cannot be accurately assessed.

This study presents some limitations: first, its retrospective design and the relatively limited number of included patients. Second, an exhaustive radio-pathological correlation could not be investigated because of the retrospective methodology of the pathologic evaluation and the nonavailability of liver explants during the study period. Third, the heterogeneity of IAT performed using different approaches (DT/BT) may be another limitation to understanding the specific prognostic impact of HVI regarding their different histological effects [50,51,52,53]. Zori et al. [47], through a series of 65 liver-transplanted patients, showed a significantly lower rate of microvascular invasion in the group treated by TARE as a BT compared with the TACE-treated patient group. In the present study, the TARE/TACE ratio was 8/55, which may have tempered the difference in the therapy-specific impact on patient outcomes.

## 5. Conclusions

The detection of an HVI on per-interventional imaging during IAT for HCC is a frequent occurrence, likely increased by the high sensitivity of CBCT. This study demonstrated the absence of any significant histological or prognostic impact of detected HVIs in HCC patients treated by IAT. These data may indicate that the treatment plan during IAT should not be impacted or modified in response to HVI detection. Additional prospective studies with larger series are needed to assess more accurately the histopathological nature of these HVIs by a radiological–anatomopathological correlation and their actual impact on patient outcomes.

## Figures and Tables

**Table 1:** Liver MRI examination protocol.

Sequence	ET (ms)	RT (ms)	Flip Angle (°)	Slice Thickness (mm)	Echo Length Train
Breath-hold fat-suppressed TSE T2-weighted sequence	2600	83	129	5	23
Breath-hold HASTE T2-weighted sequence	1000	118	120	3.5	156
Breath-hold in-phase and out-of-phase T1-weighted sequences	131	2.43–3.69	70	5	NA
Fat-suppressed T1-WI sequences	117	2.78	70	5	NA
Diffusion-weighted imaging single-shot, spin-echo-planar imaging (3 b-value 50, 400 and 800 s/mm^2^)	2500	138	150	5	23
Dynamic breath-hold 3D VIBE T1-weighted sequences	3.31	1.25	15	3	NA

Abbreviations: TSE: turbo spin-echo; HASTE: half-Fourier acquisition single-shot; NA: not applicable; ET: echo time; RT: repetition time.

**Table 2:** Epidemiologic, clinical, and biological characteristics of included patients.

Characteristics	Frequency/Mean (or Median) ± SD
***Sex***	
Male	50 (79.4%)
Female	13 (20.6%)
***Age (YO)***	59 ± 7 (33–69)
***Underlying hepatopathy***	
Alcohol abuse	42 (66.7%)
Chronic viral hepatitis B	9 (14.3%)
Chronic viral hepatitis C	6 (9.5%)
MASH	6 (9.5%)
***Child Pugh score***	
A	36 (57.2%)
B	16 (25.4%)
C	11 (17.4%)
***BCLC stage (before LRT)***	
0 or A	46 (73%)
B	16 (25.4%)
C	1 (1.6%)
***MELD score***	11.33 ± 5.25 (6–22)
***Pre-liver-transplantation AFP level (ng/mL)***	90 ± 170 (1–830)
***AFP score (when available)***	1.7 ± 1.38 (0–4)

Abbreviations: AFP: alpha-fetoprotein; BCLC: Barcelona Clinic Liver Cancer group; LRT: Loco-regional therapy; MELD: Model for End-Stage Liver Disease; MASH: metabolic dysfunction-associated steatotic liver disease.

**Table 3:** Pre and per-interventional imaging analysis results.

		Group *Incidentaloma+*			Group *Incidentaloma−*		
Patients (n)		36 (57.1%)			27 (42.9%)		
Nodules		**PII**	**CBCT**	***p*-value**	**PII**	**CBCT**	***p*-value**
	Number (median, [Q1, Q3])	2 (1, 5)	4 (3, 8)	<0.001	2 (1, 3)	2 (1, 3)	0.07
	Size of the largest nodule	23.8 ± 13 mm	24.6 ± 13 mm	0.22	29.8 ± 12 mm	30.4 ± 12 mm	0.08

Abbreviations: PII: pre-interventional imaging; CBCT: cone beam computed tomography.

**Table 4:** Subgroup analysis according to the IAT therapeutic intention (downstaging or bridging therapy).

Subgroup “Bridging Therapy”		Subgroup “Downstaging Therapy”	
*n* = 49 (77.8%)		*n* = 14 (22.2%)	
Prognostic Parameter	*p*-Value	Prognostic Parameter	*p*-Value
Tumor recurrence	0.052	Tumor recurrence	0.85
Overall survival	0.23	Overall survival	0.52

## Data Availability

The datasets used and/or analyzed during the current study are available from the corresponding author on reasonable request.
